# A Stability Study of [Cu(I)(dmby)_2_]TFSI in Biopolymer-Based Aqueous Quasi-Solid Electrolytes

**DOI:** 10.3390/gels11080645

**Published:** 2025-08-14

**Authors:** Giulia Adriana Bracchini, Elvira Maria Bauer, Claudia Mazzuca, Marilena Carbone

**Affiliations:** 1 STARTNETICS—Department of Chemical Science and Technologies, Tor Vergata University of Rome, 00133 Roma, Italy; giulia.adriana.bracchini@uniroma2.it; 2 Institute of Structure of Matter-Italian National Research Council (ISM-CNR), c/o Area della Ricerca di Roma1, Strada Provinciale 35d n. 9, Montelibretti, 00010 Rome, Italy; elvira.bauer@ism.cnr.it; 3 Department of Chemical Science and Technologies, Tor Vergata University of Rome, 00133 Roma, Italy; claudia.mazzuca@uniroma2.it

**Keywords:** [Cu(I)(dmby)_2_]TFSI, quasi-solid electrolytes (QSEs), κ-carrageenan, galactomannan, stability

## Abstract

In the field of advanced electrical energy conversion and storage, remarkable attention has been given to the development of new, more sustainable electrolytes. In this regard, the combination of redox shuttles with aqueous bio-polymer gels seems to be a valid alternative via which to overcome the typical drawbacks of common liquid electrolytes such as corrosion, volatility or leakage. Despite the promising results obtained so far, redox-active species such as bis(6,6′-dimethyl-2,2′-bipyridine)copper(I) trifluoromethanesulfonylimide, ([Cu(I)(dmby)_2_]TFSI), still present inherent challenges associated with their poor water solubility and oxidative lability, which prevents their employment in cheap and sustainable aqueous electrolytes. The present study investigates the stabilization of the Cu(I) complex ([Cu(I)(dmby)_2_]TFSI) within two natural hydrogels based on the biopolymers κ-carrageenan and galactomannan, using ZnO nanoparticles as gelling agents. These eco-friendly and biocompatible systems are proposed as potential matrices for quasi-solid electrolytes (QSEs), offering a promising platform for advanced electrolyte design in electrochemical applications. Both hydrogels effectively stabilized and retained the redox species within their networks. In order to shed light on distinct stabilization mechanisms, complementary FTIR and SEM analyses were relevant to reveal the structural rearrangements, specific to each matrix, upon complex incorporation. Furthermore, thermogravimetric analysis confirmed notable thermal resilience in both systems, with the galactomannan-based gel demonstrating enhanced performance. Altogether, this work introduces a novel strategy for embedding copper-based redox couples into gelled electrolytes, paving the way toward their integration in real electrochemical devices, where long-term stability, redox retention, and energy conversion efficiency are critical evaluation criteria.

## 1. Introduction

The development of sustainable energy technologies increasingly depends on the design of functional materials that combine high performance with environmental compatibility. In this context, quasi-solid electrolytes (QSEs) based on hydrogels represent a promising class of materials for electrochemical systems, and are capable of replacing traditional liquid electrolytes that typically employ organic aprotic solvents such as acetonitrile or propylene carbonate [1,2,3].

Although the substitution of organic solvents with water-based electrolytes represents a step towards more sustainable devices, quite often synthetic organic polymers such as polyethylene oxide (PEO), polyacrylamide (PAM) or polyvinylidene fluoride (PVDF) are employed in QSEs [4]. However, these synthetic polymers do not undergo microbial degradation, thus constituting an environmental problem at the end of their life-cycle. For this reason, scientists are now focusing on cheap and abundant bio-polymers for the development of more sustainable and “green” QSEs. The integration of eco-friendly bio-polymers into QSEs combines the benefits of aqueous media, with structural integrity, ionic mobility, and reduced volatility compared to conventional liquid-phase electrolytes [5,6,7,8]. Their use is particularly attractive in photovoltaic applications such as dye-sensitized solar cells (DSSCs), the batteries of supercapacitors, where the electrolyte plays a central role in facilitating charge transport and ensuring long-term device stability [9,10,11,12,13,14].

In this framework, hydrogels have attracted much attention in recent decades. Hydrogels are based on a network of hydrophilic polymers that can swell in liquid without dissolving, retaining a large amount of water within their three-dimensional structure. Within the hydrogel matrix, ions exhibit high mobility, which can ensure efficient energy conversion. Remarkably, hydrogel-based QSEs have demonstrated comparable or even superior performance to their liquid counterparts, with the added advantage of enhanced long-term stability [8]. The gel network structure significantly influences DSSC performance, as the degree of cross-linking during synthesis affects both the morphological and electrical properties of the QSE, including ionic conductivity and energy conversion efficiency [15].

Among the bio-polymers suitable for the construction of hydrogel QSEs, polysaccharides such as xanthan gum, cellulose or carrageenan have been widely explored [12]. For example, Galliano and co-workers proposed an aqueous xanthan gum QSE containing a iodide/triiodide redox mediator whose application in air-sealed and air-stored TiO_2_-D131 dye-based DSSC reached an efficiency of 2.7% [16]. The substitution of iodide/triiodide with bulkier cobalt bipyridine complexes as redox shuttles by the same research group pushed the efficiency of MK2-sensitized TiO_2_ photoelectrodes up to 4.47% under 1 sun illumination [17]. Quite recentlyn Kandhasamy et al. blended properly modified copper redox shuttles in an aqueous pectin-2-hydroxyethylcellulose matrix [18]. The gel was employed as an electrolyte, after the addition of limonene extracted from orange peel, in a TiO_2_/N3 dye-based DSSC achieving a PCE of 4.4%. Quite promising also are polysaccharides derived from algae such as carrageenans for building up a polymer matrix suitable to hold in aqueous electrolytes. In this anionic biopolymer, the sulfate ester and hydroxyl groups allow cations to be bonded to the polymer back bone, improving the ionic conductivity by an electron hopping mechanism. These interactions can be further enhanced by chemical modifications of the bio-polymer, such as carboxymethylation, which boost its ionic conductivity from 5.34 × 10^−7^ S cm^−1^ towards 3.4 × 10^−5^ S cm^−1^ [19]. The introduction of new coordination sites in this anionic polymer makes it possible to further increase the conductivity by doping with suitable salts. In this regard, Torres et al. investigated the possibility of enhancing ionic motion by doping a carboxymethyl κ/ι-carrageenan gel with NH_4_I and glycerol. In the resulting solid aqueous electrolyte, conductivities around 3.9 × 10^−3^ S cm^−1^ could be reached, values which lie close to solid electrolytes based on synthetic polymers [20]. However, these results did not outperform the carboxymethyl κ-carrageenan electrolyte proposed by Bella et al. The latter research group doped the modified bio-polymer with NaI in the presence of the plasticizer ethylene carbonate, whose optimum amounts have been determined by a chemometric approach. The optimized system contained 45% NaI and 30 wt% plasticizer, reaching exceptional conductivities around 5.53 × 10^−2^ S cm^−1^. After the sublimation of iodine into the polymer gel matrix, the quasi-solid electrolyte membrane was contacted with a TiO_2_-N179 dye photoanode, reaching conversion efficiencies of 2.06% under 1 sun illumination, with an efficiency drop of only 6% after 250 h [19].

Besides a stable gel network, the most important component in QSE remains the redox mediator. Quasi-solid electrolytes based on iodine redox shuttles have been widely reported in the literature [21], but the traditional iodide/triiodide (I^−^/I_3_^−^) couple, though effective, presents well-known limitations, including corrosivity, light absorption in the visible spectrum, and stability issues [22,23]. To address these limitations, cobalt-based complexes were initially introduced as promising alternatives [24,25], followed more recently by copper-based complexes, which offer additional advantages in terms of tunability, reduced cost, and lower environmental impact.

Copper-based redox couples, such as [Cu(dmby)_2_]TFSI (where dmby = 6,6′-dimethyl-2,2′-bipyridine), have emerged as attractive alternatives due to their favorable redox properties, lower toxicity, and compatibility with low-cost and CRM-free device architectures [26]. However, the poor solubility of such complexes in water poses a significant challenge to the formulation of aqueous or water-based electrolytes.

This study addresses this challenge by developing and characterizing two novel hydrogels to be used as matrices for QSE assembly, derived from natural biopolymers as stable, eco-friendly hosts for incorporating [Cu(dmby)_2_]TFSI. The first is based on galactomannan, while the second employs κ-carrageenan as the hydrogel matrix. Both systems incorporate zinc-derived nanoparticles synthesized via a green, energy-efficient process. In addition to functioning as gel-forming agents [27], these nanoparticles retain intrinsic antimicrobial properties, which may enhance the functional stability and longevity of the hydrogels matrix [28,29]. Inorganic nanoparticles are often added to electrolytes as gelators with the intention to form a physically or chemically cross-linked network able to suppress leakage problems and solvent evaporation. Hence, their action will increase electrolyte stability, and may reduce charge transfer rates and conversion efficiencies. The addition of inorganic nanoparticles such as ZnO can overcome this problem because the resulting 3-D network may facilitate charge transfer via the Grotthus-type ion exchange mechanism, especially when small amounts are added. The positive effect of ZnO NPs as a gelator in a QSE has been previously proven in ZnO-based DSSCs where the ZnO NPs provided efficient charge transfer channels, improving the short-circuit current density, open-circuit voltage and electron lifetime [30]. Furthermore, interaction between ZnO nanoparticles and the selected polysaccharides shall reduce the crystallinity of the polymer matrix, another factor affecting charge transfer negatively. The incorporation of ZnO nanoparticles into our systems was largely inspired by our previous work [27] on the in situ formation of ZnO NPs in galactomannan-based QSEs. The state-of-the-art aqueous QSE-DSSC device assembled after the addition of the I^−^/I_3_^−^ redox mediator showed an unprecedented open-circuit voltage (VOC) of up to 750 mV, accompanied by a power conversion efficiency of 2%.

On this basis, the hydrogels presented here not only support the dispersion and stabilization of the copper(I) complex, but also introduce advantageous material properties such as non-volatility, mechanical integrity, and thermal resilience [31].

A central objective of this work is to elucidate the structure–property relationships governing the performance and stability of these hybrid hydrogel systems. The materials were comprehensively characterized through thermogravimetric analysis (TGA), Fourier-transform infrared spectroscopy (FTIR), and scanning electron microscopy (SEM). These techniques were used to investigate the interaction between the copper complex and the polymeric matrix, as well as the resulting morphological and thermal behavior of the hydrogels.

The temporal stability of the [Cu(dmby)_2_]TFSI complex within each matrix was assessed through visual monitoring over one day, with particular attention paid to the preservation of the Cu(I) oxidation state. The results indicate that both hydrogel formulations successfully stabilize the copper complex and exhibit favorable thermal profiles, with the galactomannan-based matrix showing enhanced stability under the tested conditions.

By integrating spectroscopic, thermal, and morphological data, this study contributes fundamental insights into the stabilization mechanisms of redox-active species within soft polymeric networks. The findings support the continued development of hydrogel-based quasi-solid electrolytes, not only for DSSCs but more broadly as functional materials in electrochemical and energy storage applications.

## 2. Results and Discussion

### 2.1. Temporal and Thermal Stability

To assess the ability of biopolymer-based hydrogels to incorporate and stabilize a water-insoluble redox-active species, we investigated the behavior of [Cu(I)(dmby)_2_]TFSI within two distinct nanocomposite gel matrices: κ-carrageenan-ZnO (CARZ) and galactomannan-ZnO (GMZ).

The UV-Vis spectra of the CARZCu gel over time (up to about 9 h) reveal a broad absorption band between 400 and 640 nm, and this feature is comparable to the absorption reported by Saygili et al. for the same copper complex in an acetonitrile solution at a concentration of 0.5 mM [26].

**Figure 1:**
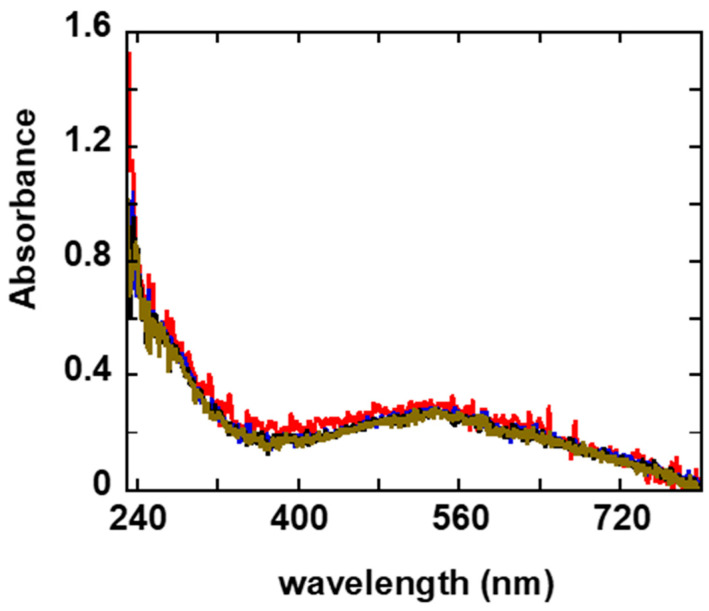
UV-vis spectra of the [Cu(dmby)_2_]TFSI complex inside the CARZ gel as a function of time: immediately after addition to the gel (red), and after 120 (blue), 240 (black) and 510 (brown) minutes.

The CARZCu spectra do not show significant variations among themselves (Figure 1); in fact, no peak shifts in either intensity or position are observed. This result indicates, first of all, that the copper complex did not undergo oxidation, and that its molecular structure was coordinated to the polymer network of the gel, preventing both precipitation and aggregation.

**Figure 2:**
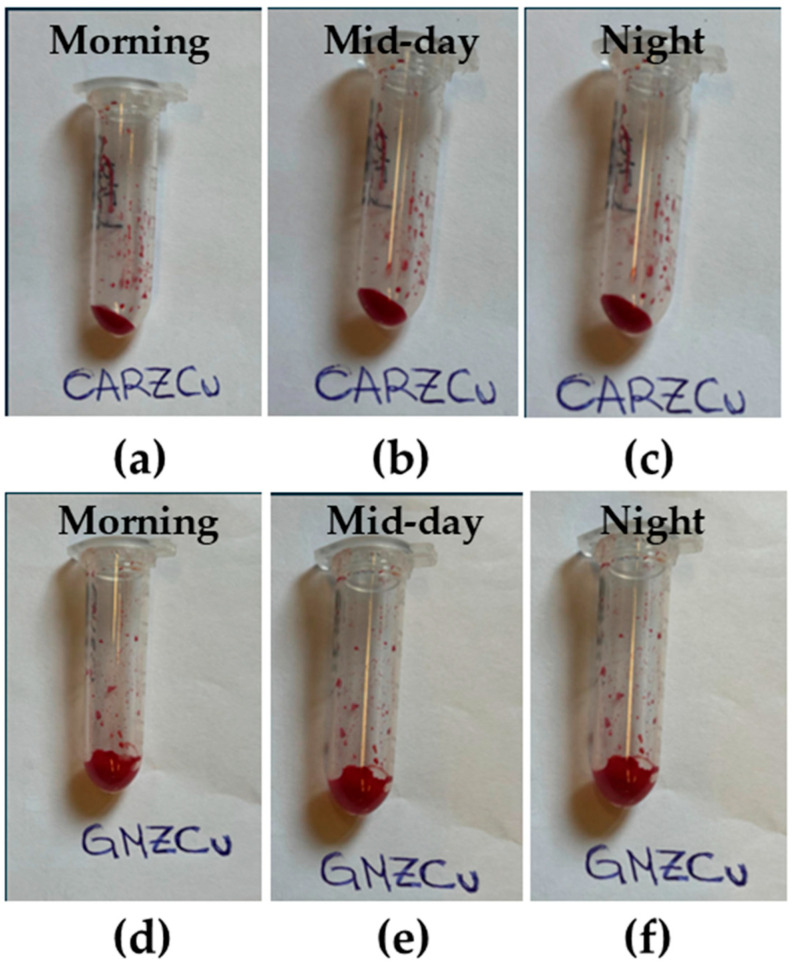
Photo of CARZCu and GMZCu after preparation (**a**,**d**), at mid-day (**b**,**e**) and at the end of the day (**c**,**f**).

The incorporation and short-term stability of the complex in these systems were also evaluated through photographic documentation (Figure 2). This method was used to monitor potential macroscopic changes—such as discoloration, aggregation, or precipitation—that could indicate the instability of the complex within the gels. Images taken in the morning, mid-day, and evening of the same day show that both the CARZCu and GMZCu samples retained a uniform, intense red coloration, with no visible changes in color intensity or distribution. The absence of any signs of phase separation or precipitation suggests that the Cu(I) complex remains stably incorporated in both systems over the observed time frame.

The poor aqueous solubility of Cu-based complexes has largely restricted electrochemical studies of Cu redox couples to liquid electrolytes using conventional organic solvents [32,33]. Only recently, the in situ gelation of electrolytes containing [Cu(I)(dmby)_2_]TFSI, by blending one or two polymers (PVDF-HFP) into liquid electrolytes at appropriate ratios, has been investigated, resulting in quasi-solid-state DSSCs achieving over 36% efficiency [34]. However, even in these studies, acetonitrile was still the solvent employed to dissolve the redox species. In contrast, the present work effectively overcomes the challenge of the complex’s inherently low aqueous solubility by dispersing it within ZnO-containing biopolymer hydrogels, which provide a stable medium for its retention, without the additional use of traditional organic solvents.

One crucial aspect to assess in our systems, as potential alternative matrices for electrolytes in DSSCs or for electrochemical applications in general, is their thermal stability. In this context, we specifically examined how the incorporation of a Cu complex affects the thermal resistance and degradation behavior of our hydrogels.

**Figure 3:**
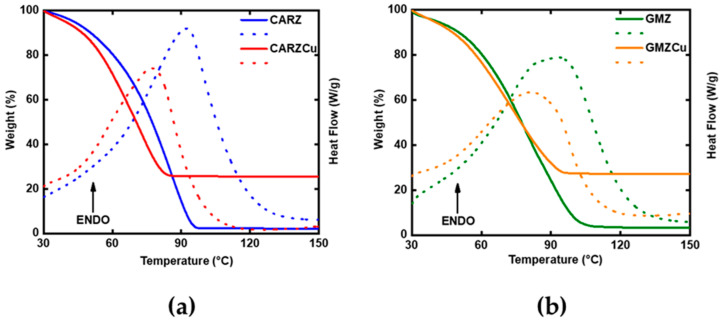
TGA (solid lines) and DSC (dashed lines) curves of the CARZ and CARZCu samples (**a**), GMZ and GMZCu (**b**).

The TGA of the CARZ gel, shown in Figure 3a, reveals thermal degradation over a broad temperature range, starting at 30 °C and reaching a plateau around 97.5 °C, with a residual mass of only 2% of the initial weight. Upon incorporation of the Cu complex, the resulting curve indicates a reduced resistance to mass loss compared to the pure CARZ gel: although degradation still begins at 30 °C, it stabilizes earlier, at around 84 °C, with a residual mass of 25.6%.

Considering that the temperature range used in this analysis does not induce the degradation of either κ-carrageenan or the Cu complex [35,36], we hypothesize that the mass loss observed for the CARZ sample between 30 °C and 84 °C is solely attributable to the release of water molecules retained within the gel matrix.

As can be seen from the comparison of the Differential Scanning Calorimetry (DSC) curves for the two systems (dashed line), the gel–sol transition [37] followed by the expulsion of water from the hydrogel is an endothermic process, indicated by a broad peak at 92.5 °C for CARZ and 77 °C for CARZCu, which shifts to lower temperatures and shows a reduced peak area following the addition of the Cu complex.

The TGA and DSC analysis of the galactomannan-based gel, GMZ, and its Cu-containing counterpart, GMZCu, yielded results similar, though not identical, to those observed in the previously discussed systems. The GMZ gel, in the absence of the Cu complex, undergoes thermal degradation between 30 °C and 109 °C, eventually stabilizing with a residual mass of 3.3%.

Upon incorporation of the Cu complex, a steeper slope is observed in the thermogravimetric curve within the 30–77 °C range, compared to GMZ, followed by a more gradual slope between 77 °C and 95 °C. The curve then levels off, reaching a plateau, with a final residual mass of 27%, which can once again be primarily attributed to the Cu complex, known for its thermal stability, within this temperature range.

Also, in this galactomannan-based hydrogel system, the incorporation of the Cu complex results in an accelerated mass loss, associated with the likely facilitated release of structurally bound water from the gel matrix.

The DSC curves show a shift in the endothermic peak associated with the gel–sol transition and the release of water from the network to lower temperatures (from 92 °C to 82 °C for GMZ and GMZCu, respectively) and, also in this case, a reduced peak area.

It is worth noting that, although the addition of the Cu complex leads to a decrease in thermal stability (as evidenced by TGA) in both hydrogel systems studied, the galactomannan-based hydrogel appears to be less affected by the introduction of the Cu complex. At 75 °C, the difference in residual mass between CARZ and CARZCu is 16%, whereas for GMZ and GMZCu it is only 3%.

From a calorimetric perspective (DSC), both hydrogel systems exhibit the same trend: after the incorporation of the Cu(I) complex, the area of the endothermic peak decreases, indicating a reduced enthalpic contribution to water expulsion.

In previous studies, such as that by Shibayama et al. [38], the endothermic peak observed during the heating of a hydrogel phase has been microscopically associated with a decrease in the number of hydrogen bonds between the hydrophobic groups of the monomer units and a reduction in the number of water molecules in their vicinity (dissociation of hydrophobic interactions).

The heat absorption required to reduce the number of hydrogen bonds in the contracted hydrogel depends on the degree of crosslinking in the three-dimensional polymer network that characterizes the hydrogel. Therefore, the behavior observed in our systems suggests that the three-dimensional networks in both CARZCu and GMZCu are altered compared to those of CARZ and GMZ, respectively.

On one hand, the incorporation of the Cu(I) complex into the hydrogel matrices appears to facilitate a more rapid disruption of the intermolecular forces responsible for network formation. On the other hand, the shift in the endothermic peak to lower temperatures may also indicate enhanced ion mobility within the polymer matrix.

This is consistent with the findings reported for QSE plasticized chitosan biopolymer electrolytes [39], where the endothermic peak observed upon heating from room temperature to 150 °C is attributed to the gel–sol transition. During this transition, the polymer shifts from a rigid to a rubbery state, enabling increased segmental motion prior to reaching its melting temperature.

It is important to emphasize that below the gel–sol transition temperature, polymer chains remain rigid and immobile, whereas above this temperature, the polymer becomes flexible, allowing greater chain mobility and, consequently, enhanced ionic transport. 

### 2.2. Characterization

The spectroscopic characterization of the systems proposed in this study aims to detect any structural modifications induced by the incorporation of the copper complex into the gel matrix. To this end, the FTIR spectrum of the pure complex [Cu(I)(dmby)_2_]TFSI was analyzed and compared with the FTIR spectra of the two gels, recorded before and after the introduction of the complex, at a final concentration of 0.2 M, which was deemed suitable for DSSC applications [40].

Concerning the FTIR absorption spectrum of [Cu(I)(dmby)_2_]TFSI, the characteristic vibrations of the -dmby ligands can be identified, including the C–H stretching of aromatic rings between 3100 and 2900 cm^−1^, the bands at 1550–1600 cm^−1^ corresponding to C=C and C=N stretching within the aromatic systems, and the signal at 780 cm^−1^ associated with ring deformation [41,42]. Regarding the TFSI counter ion, the asymmetric and symmetric stretching modes of the –SO_2_ groups are observed at 1350 and 1130 cm^−1^, respectively. The strong peak at 1195 cm^−1^, accompanied by a shoulder at 1225 cm^−1^, is attributed to the asymmetric stretching of the –CF_3_ group. The asymmetric S–N–S stretching appears at 1060 cm^−1^, and the SO_2_ bending vibration is found at 618 cm^−1^ [43].

**Figure 4:**
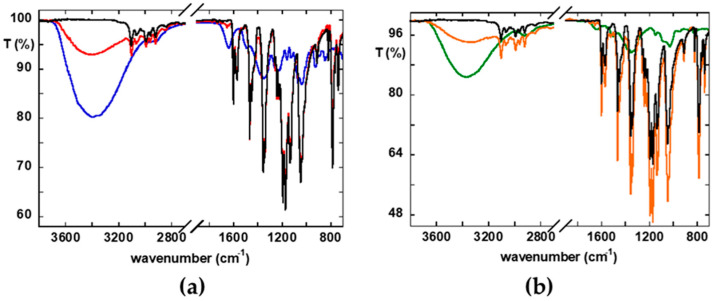
FTIR of (**a**): CARZ (blue), CARZCu (red), [Cu(I)(dmby)_2_]TFSI (black) and of (**b**): GMZ (green), GMZCu (orange), [Cu(I)(dmby)_2_]TFSI (black).

In the FTIR spectrum of CARZ (Figure 4a), the broad band at 3375 cm^−1^, attributed to the symmetric stretching of –OH groups from water (which constitutes approximately 99% of the hydrogel), remains unchanged and easily identifiable when comparing the spectra before (blue trace) and after (red trace) copper complex addition.

Several characteristic bands of κ-carrageenan are clearly visible. Among the carrageenan-specific bands, the following can be identified in the pristine sample: the broad –OH stretching band centered around 3370 cm^−1^; the asymmetric and symmetric C–H stretching modes at 2964 and 2899 cm^−1^, respectively; the S=O or C–O stretching of ester sulfate groups at 1225 and 1155 cm^−1^; and the C–O stretching of primary alcohols at 1065 cm^−1^. Particularly relevant are two characteristic bands of carrageenan observed at 921 and 845 cm^−1^, attributed to the C–O–C stretching of 3,6-anhydro-D-galactose and the bending vibrations of ester sulfate groups, respectively (peak assignments are consistent with previous studies [37,43,44]). However, these features are no longer distinguishable in the spectrum of CARZCu due to the overlap with intense absorption signals of the Cu(I) complex. Indeed, the FTIR spectrum of CARZCu is dominated by the characteristic vibrational peaks of the Cu(I) redox species. The nearly complete coincidence of the vibrational features of the CARZCu with the pristine copper complex clearly indicates the conservation of its chemical nature in the aqueous gel environment. After the immobilization of the copper complex in the biopolymer matrix, neither peak shape changes nor slight shifts in its characteristic peak positions are detectable. In variance, a gas-phase IRMPD investigation of isolated Cu(II) bipyridil and Cu(I) bipyridil ions, i.e., [Cu(by)_2_]^2+^ and [Cu(by)_2_]^+^, showed a 10 cm^−1^ red shift in the out-of-plane C-H bending vibrations located around 750 cm^−1^ and C-H in-plane bending around 1160 cm^−1^ upon charge reduction [45]. The absence of any variances in our sample may suggest the stabilization of the oxidation state of the copper complex through interactions, for instance, with the –SO_3_^−^ functional groups of the carrageenan matrix that are located on the outer region of the linear carrageenan backbone [46] and are therefore more accessible for metal coordination.

In general, the introduction of the [Cu(I)(dmby)_2_]TFSI complex induces a partial rearrangement of the native three-dimensional network between the carrageenan polymer chains [47,48], likely as a result of specific interactions between Cu(I) and the –SO_3_^−^ groups of the polymer.

In the case of the galactomannan-based gel (Figure 4b), the analysis of the IR spectra follows a similar approach. The characteristic signals of galactomannan, clearly identifiable in the spectrum of the GMZ sample, include the stretching of –CH_2_ groups below 3000 cm^−1^, several bands in the 1100–1000 cm^−1^ region, and multiple shoulders below 800 cm^−1^, consistent with previous literature [49]. These bands are not clearly visible in the spectrum of the GMZCu sample, due to overlapping with the more intense signals of the Cu complex. The near-complete overlap between the spectra of GMZ and GMZCu suggests that any possible vibrational shifts in galactomannan functional groups, potentially arising from interactions with the Cu(I) complex, are minimal and insufficient to alter the peak shapes, attributed to the Cu complex alone.

In summary, in both cases, at this concentration of the redox species dissolved in the gels, FTIR spectra are predominantly characterized by the highly intense absorption bands of the Cu(I) complex. Nevertheless, its immobilization in the two hydrogels does not imply changes in the spectral characteristics of the copper species, suggesting an interaction between the biopolymer and the metal center of the complex.

However, the incorporation of the Cu(I) complex into the carrageenan matrix is believed to promote a partial disintegration or rearrangement of the native three-dimensional network, which may be more pronounced than that observed in the galactomannan-based gel. This difference is primarily attributed to the presence of sulfonate groups (–SO_3_^−^) within the molecular structure of carrageenan, which are known to strongly interact with Cu(I) [50,51]. Although the metal is already coordinated, it can still act as a bridging site between adjacent polymer chains. The linear nature of carrageenan chains [52] further facilitates their approach and alignment, thereby favoring intermolecular interactions and giving a new possible network arrangement and entanglement. In the review by Liu et al. [53], it is clearly stated that the greater the polymer chain entanglement, the higher the flexibility and deformability of a hydrogel, which becomes capable of quickly returning to its original state after being subjected to external stress or deformation. In some cases, such hydrogels can even retain high levels of ionic conductivity. Therefore, in the present case of the κ-carrageenan hydrogel, we can hypothesize that the incorporation of the Cu(I) complex may promote the entanglement of biopolymer chains, which can reduce the breakage of chemical bonds by avoiding stress concentration and rapidly dissipating the applied stress. This, in turn, minimizes energy loss and ultimately leads to the formation of a hydrogel with enhanced mechanical properties.

In contrast, galactomannan lacks –SO_3_^−^ groups and contains only hydroxyl groups (–OH) capable of weakly interacting with Cu(I). Moreover, its branched molecular architecture [54] introduces significant steric hindrance, which limits the ability of the chains to approach each other closely and form effective interactions. 

### 2.3. SEM Analysis

The morphological analysis of the two hydrogels proposed in this study allowed us to characterize the presented systems with the aim of integrating our understanding of the effects caused by the introduction of the Cu complex within the two gel matrices.

**Figure 5:**
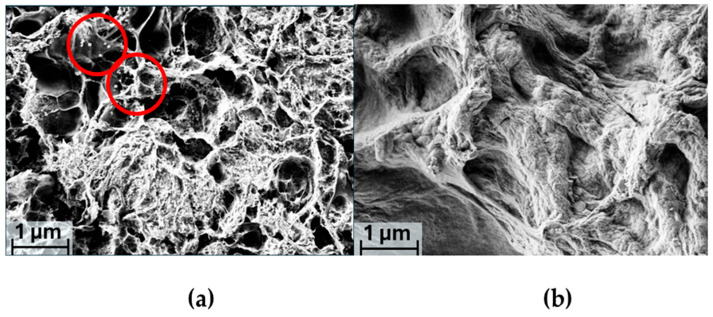
SEM images of CARZ (**a**) and CARZCu (**b**) at 20.00 KX magnification. The red circles indicated the white ZnO agglomerates.

The 1 μm magnification (20.00 Kx) of the CARZ sample, shown in Figure 4a, highlights a spongy structure with distinct pores evenly distributed throughout. This type of porosity and the interconnections between the voids are consistent with a cross-linked polymer network, typical of a κ-carrageenan-based gel [55]. The ZnO particles, synthesized within the hydrogel, are clearly visible due to a higher contrast, owing to their higher atomic number compared to the organic matrix. They appear as small white agglomerates, distributed in a non-homogeneous manner (circled in Figure 5a). This SEM image further reveals a fibrous microstructure at the local level, suggesting a well-formed gel, due to the strong interaction between the polymer chains. Additionally, it is evident that the ZnO particles are not only present on the surface of the pores but are also well embedded within the matrix.

The SEM images of the CARZCu sample (Figure 5b), when compared with those previously discussed, reveal significant differences. In this case, the pores are less visible and the white agglomerates associated with ZnO particles are no longer observed. The Cu complex appears to coat the polymer matrix and the embedded ZnO particles in a completely homogeneous manner, leading to the stiffening and closer packing of the polymer chains. Indeed, extended areas of nearly linearly aligned polymer chains are visible in the CARZCu sample, pointing towards a less rigid but more compact network in accordance with the FTIR and DSC measurements. As just mentioned, enhanced interaction between the linear polymer chains should also improve the mechanical properties (tensile strength, elastic modulus and elongation at break) of the resulting hydrogel. In fact, the decrease in tensile strength and elastic modulus has been ascribed to the weaker interfacial interactions between the linear polymer chains in κ-carrageenen/ZnO films derived from hydrogels [28]. Interestingly, the elongation of the break of the inspected nanocomposite films increased as the uniform distribution of NPs did not interrupt the movement of the chains. On the contrary, the uniform coating and denser morphology observed in our CARZCu sample leaves us to expect an improvement in the mechanical properties of the hydrogel with respect to CARZ. 

The homogeneous coating by the copper complex further confirms the FTIR analysis results regarding the ability of the κ-carrageenan-ZnO gel to stabilize and retain the electrolyte in the matrix through interactions between the copper ion and the functional groups of κ-carrageenan. Interestingly, the excellent redox mediator retention capacity of carrageenan-based gels has been previously proven by Crumbliss et al. in the case of cationic ruthenium(II) and cobalt(III) tris(2,2′-bipyridine) complexes [56]. The diffusion characteristics of the cationic redox couples in the hydrogels outperformed other immobilization matrices such as NAFION, most likely due to the fact that the hydrogel structure is mostly water, allowing the high physical mobility of the complexes. Charge propagation was dominated by physical diffusion accompanied by electron hopping, i.e., electron exchange between charged redox species electrostatically bound to functional groups on the polymer backbone. Similar results may be expected also in the case of our nanocomposite gel, taking into consideration that the diffusion coefficient of the copper complex is two times higher than the cobalt complex [57].

For the GMZ sample, a SEM image acquired at different magnification (10.00 Kx) was selected, as this setting more effectively highlights the development of porosity in the system, enabling a clearer comparison with the carrageenan-based sample.

**Figure 6:**
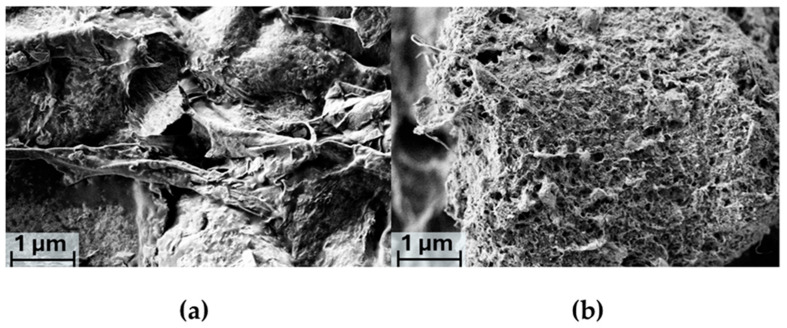
SEM images of GMZ (**a**) and GMZCu (**b**) at 10.00 Kx magnification.

The 1 μm magnification in Figure 6a reveals a markedly irregular, lamellar structure. The porosity appears less developed compared to the carrageenan-based system, with larger, non-interconnected pores that remain open between the folds of the matrix. In this case, ZnO particles are not clearly distinguishable at either magnification, likely due to being masked by the rough surface morphology.

The incorporation of [Cu(I)(dmby)_2_]TFSI promotes the formation of a more regular microstructure, as evidenced by the well-distributed and interconnected porous morphology observed in Figure 6b, and these observations suggest that [Cu(I)(dmby)_2_]TFSI is uniformly distributed throughout the galactomannan polymer network. 

Considering the homogenous distribution of the redox species in our sponge-like nanocomposite, the sufficiently high diffusion of the redox species in the hydrogels may be expected. However, it is worth noting that nearly solid Cu(I)/Cu(II) bipyridyl redox shuttles behave as hole conductors in which charge is probably transferred by percolation-hopping. This behavior had surprisingly positive effects in so-called “Zombie cells” (DSSCs in which the electrolyte solution is nearly absent) [57]. In fact, upon slow solvent evaporation in PEDOT/PEDOT-based symmetric dummy cells soaked with the copper redox mediator, intermediate charge-transfer stages in which the physical mobility of the redox shuttle and electron hopping contribute equally to the electrolyte performances have been proposed. The inspection of our freeze-dried GMZCu sample indicates not only a porous network favorable for solvent infusion but also uniform coating with the copper complex associated with higher thermal stability, which may provide an interesting platform for the investigation of QSEs, as well as for solid electrolytes.

## 3. Conclusions

In this work, we developed two hydrogel systems—one based on κ-carrageenan and the other on galactomannan biopolymers—both incorporating ZnO as sustainable alternatives to the conventional organic solvents typically used in electrolytes for electrochemical systems.

Within these matrices, we successfully dispersed the redox-active species [Cu(I)(dmby)_2_]TFSI, achieving the stabilization of this complex, which is otherwise poorly soluble in water and prone to degradation. The resulting formulations maintained the complex in a chemically stable, oxidation-resistant state.

Thermal analysis revealed that the galactomannan-based gel outperformed its carrageenan counterpart in the 25–150 °C range. TGA showed no significant destabilization upon Cu complex incorporation into the galactomannan gel, unlike in the carrageenan system.

IR spectroscopy and SEM provided further insights into the structural response of the hydrogels. While the Cu complex induces some network reorganization in both systems, this effect is less pronounced in the galactomannan gel. Its branched structure introduces steric hindrance, preserving –OH groups for hydrogen bonding and enhancing water retention.

In contrast, carrageenan’s linear chains align more readily. Here, the Cu complex likely promotes inter-chain interactions, reducing hydrophilic sites and accelerating water loss, as reflected in the higher TGA mass loss.

These results support the potential use of biopolymer-based hydrogels as quasi-solid electrolytes (QSEs). The galactomannan–ZnO system, in particular, combines favorable thermal behavior with structural resilience and sustainability. Future work will explore electrochemical properties, long-term stability, and device performance, advancing these materials toward practical application in green energy technologies.

## 4. Materials and Methods

### 4.1. Materials

Zinc nitrate (Zn(NO_3_)_2_·6H_2_O, CAS No. 10196-18-6), sodium hydroxide (NaOH, CAS No. 1310-73-2), Galactomannan (CAS No. G0753) (GM), k-carrageenan (CAS No. 9000-07-1) (CAR), 6,6′-dimethyl-2,2′-bipyridine trifluoromethanesulfonyl-imide, and [Cu(dmby)_2_]TFSI (CAS No.1882083-74-0) were purchased from Sigma-Aldrich (Merck KGaA, Darmstadt, Germany). Deionized water (DI-H_2_O, 18 MΩ cm at 25 °C) was obtained with a Direct-Q 3 UV Water Purification System (Millipore, Billerica, MA, USA).

### 4.2. Gels Synthesis

The preparation of the hydrogels follows a three-step protocol, spanning a total of three days. Initially, the biopolymer of interest is dissolved in distilled water. Subsequently, ZnO nanoparticles are synthesized directly within the aqueous solution via a precipitation reaction, as part of a green synthetic approach for the production of ZnO, as gel-forming components, but also for their inherent antimicrobial activity, which could contribute to improving the functional stability and extended lifespan of the gels matrix [27]. Finally, centrifugation and successive washing steps with distilled water are performed, to obtain the desired gel phases.

With respect to the nanocomposite formulation, it is worth pointing out that under the experimental conditions chosen in this work, the gelification of the two matrices occurs only upon the in situ formation of the ZnO NP. This observation underlines the capability of the generated ZnO NPs to serve as suitable connection sites between the respective polymer chains.

To prevent the formation of mold within the gels, prior to the in situ generation of antibacterial ZnO, all glassware and tools required for the hydrogel synthesis were sterilized in distilled water at 100 °C for 1 h as a preliminary step before the three main stages of the protocol.

The complete dissolution of κ-carrageenan or galactomannan in distilled water was achieved by adding 1 g of the selected biopolymer to 100 mL of distilled water (1% *w*/*v*) and maintaining the resulting aqueous solutions at 60 °C in an oil bath under vigorous magnetic stirring for 24 h. After this period, Zn(NO_3_)_2_·6H_2_O was added directly into the same reaction flasks to reach a final concentration of 2% *w*/*v* relative to the initial solution. The mixtures were then kept under continuous magnetic stirring at 60 °C for an additional 2 h to ensure the complete dissolution of the zinc salt.

Subsequently, 100 mL of 0.1 M NaOH was added dropwise to the reaction flasks, and the resulting mixtures were left to react for 12 h at 60 °C under vigorous magnetic stirring, allowing the reaction (referred to as Reaction (1)) to proceed.

Zn(NO_3_)_2_ + 2NaOH → Zn(OH)_2_ (s) + 2NaNO_3_ (aq) → ZnO (s) + H_2_O + 2NaNO_3_ (aq), (1)

The third and final step of this synthesis procedure involves the centrifugation of the material obtained from the previous two steps at 4500 rpm and 25 °C. After centrifugation, the excess liquid is removed, and the remaining gel is collected and washed with distilled water, followed by centrifugation. This washing–centrifugation cycle is repeated three times.

The 1% carrageenan-based hydrogel containing ZnO appears as a gelatinous, milky-white mass due to the presence of finely and homogeneously dispersed zinc oxide particles within the polymeric matrix. To the touch, it feels highly hydrated, with a soft and slippery texture, suggesting low apparent viscosity and a high fraction of free water.

In contrast, the 1% galactomannan-based hydrogel containing ZnO exhibits a completely white and opaque appearance, along with a much firmer and drier texture. This gel is compact, less deformable, and more cohesive, indicating a higher apparent viscosity and a denser cross-linked network structure, capable of retaining water, in a more structured and less freely mobile form compared to the carrageenan-based hydrogel. The synthesized gels are listed in Table 1.

### 4.3. [Cu(I)(dmby)_2_]TFSI Incorporation

Approximately 0.01 g of each sample CARZ and GMZ was collected, and 0.07 g of [Cu(I)(dmby)_2_]TFSI was added to each. The resulting mixtures were initially stirred manually, then subjected to vortex mixing three times (approximately 3 min in total), alternating each vortex cycle with manual stirring. The obtained systems are listed in Table 2.

### 4.4. Temporal and Thermal Stability Analysis

To evaluate the temporal stability of the [Cu(I)(dmby)_2_]TFSI complex within the proposed hydrogels, it was verified that the complex remained uniformly dispersed throughout the gel matrix over the course of a day, without undergoing precipitation.

To this end, UV-vis spectra were acquired at hourly intervals to monitor any changes in optical absorption that could indicate instability or aggregation of the complex. UV-Vis spectra were recorded on an Agilent Cary 60, (Agilent, Billerica, CA, USA), using a 10 mm quartz cuvette, (Hellma, GmbH, Müllheim, Germany).

Photographic documentation, showing the CARZCu and GMZCu samples immediately after preparation, was performed at midday and at the end of the day.

Thermogravimetric analysis (TGA), performed using the Discovery SDT 650 instrument, (TA Instruments, New Castle, DE, USA), was carried out on all synthesized gels both before and after the incorporation of the copper complex in order to evaluate their thermal stability. The temperature program ranged from 30 °C to 150 °C at a heating rate of 10 °C/min, under a dry nitrogen flow (100 mL/min).

### 4.5. Structural and Morphological Characterization

Infrared spectra were recorded on all samples with a Shimadzu Prestige-21 FT-IR instrument (Shimadzu Corporation, Kyoto, Japan), equipped with an attenuated total reflectance (ATR) diamond crystal (Specac Golden Gate, Shimadzu Corporation, Kyoto, Japan), in the 400–4000 cm^−1^ range, with a resolution of 4 cm^−1^ and with the Thermo-Scientific instrument (model Is50) (Thermo Scientific Inc., Madison, WI, USA) in Attenuated Total Reflectance (ATR) mode using a single reflection diamond cell. Spectra were recorded from 4000 to 500 cm^−1^, averaging over 32 scans with a resolution of 4 cm^−1^. The morphology of the freeze-dried CARZ, GMZ CARZS, CARZCu, CARZSCu and GMZCu samples was determined by FE-SEM, Field Emission Scanning Electron Microscope SUPRA TM 35, (Carl Zeiss, SMT Oberkochen Germany), operating at voltages between 1.5 and 7 kV, after deposition on a silicon wafer, used as a sample holder.

## Figures and Tables

**Table 1:** List of synthesized gels; CARZ, GMZ.

Sample Label	ZnO Concentration	GM%	CAR%
CARZ	0.025 M	X	1
GMZ	0.025 M	1	X

**Table 2:** List of obtained systems; CARZCu, GMZCu.

Sample Label	ZnO Concentration	GM%	CAR%	Cu Complex
CARZCu	0.025 M	X	1	0.07 g
GMZCu	0.025 M	1	X	0.07 g

## Data Availability

The data presented in this study are available on request from the corresponding author due to ongoing research on the materials investigated.
